# 3,14-Diethyl-2,13-di­aza-6,17-diazonia­tri­cyclo­[16.4.0.0^7,12^]docosane dichloride tetra­hydrate from synchrotron radiation

**DOI:** 10.1107/S1600536813027232

**Published:** 2013-10-09

**Authors:** Dohyun Moon, Md Abdus Subhan, Jong-Ha Choi

**Affiliations:** a Pohang Accelerator Laboratory, POSTECH, Pohang 790-784, Republic of Korea; b Department of Chemistry, Shah Jalal University of Science and Technology, Sylhet, Bangladesh; c Department of Chemistry, Andong National University, Andong 760-749, Republic of Korea

## Abstract

The asymmetric unit of title hydrated salt, C_22_H_46_N_4_^2+^·2Cl^−^·4H_2_O, comprises half a centrosymmetric dication, one Cl^−^ anion and two water mol­ecules of crystallization. The structure determination reveals that protonation has occurred at diagonally opposite amine N atoms, and that the dication features intra­molecular N—H⋯N hydrogen bonds. In the crystal, a three-dimensional artchitecture is formed by O—H⋯Cl/N and N—H⋯Cl/O hydrogen bonds.

## Related literature

For background to the coordination chemistry of tetra­aza­macrocycles, see: Choi *et al.* (2010 ▶); De Clercq (2010 ▶). For the synthesis of the precursor macrocycle, see: Lim *et al.* (2006 ▶). For related structures, see: Choi *et al.* (2006 ▶, 2011 ▶).
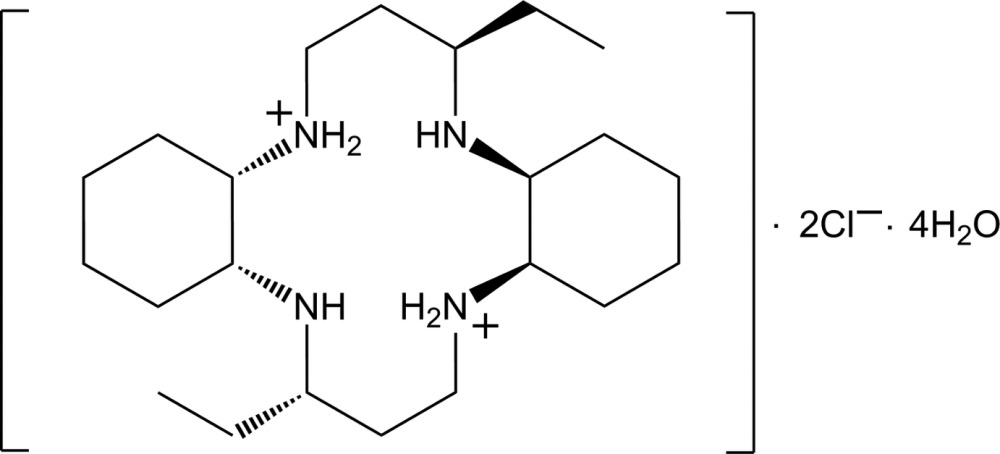


## Experimental

### 

#### Crystal data

C_22_H_46_N_4_^2+^·2Cl^−^·4H_2_O
*M* *_r_* = 509.59
Monoclinic, 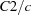

*a* = 22.122 (4) Å
*b* = 13.616 (3) Å
*c* = 10.565 (2) Å
β = 115.23 (3)°
*V* = 2878.5 (10) Å^3^
*Z* = 4
Synchrotron radiation
λ = 0.72000 Å
μ = 0.27 mm^−1^
*T* = 95 K
0.31 × 0.28 × 0.25 mm

#### Data collection

ADSC Q210 CCD area-detector diffractometer
Absorption correction: empirical (using intensity measurements) (*HKL-3000 *SCALEPACK**; Otwinowski & Minor, 1997 ▶) *T*_min_ = 0.922, *T*_max_ = 0.937
13046 measured reflections
3663 independent reflections
3446 reflections with *I* > 2σ(*I*)
*R*_int_ = 0.028

#### Refinement

*R*[*F* ^2^ > 2σ(*F* ^2^)] = 0.029
*wR*(*F* ^2^) = 0.079
*S* = 1.05
3663 reflections
178 parameters
H atoms treated by a mixture of independent and constrained refinement
Δρ_max_ = 0.46 e Å^−3^
Δρ_min_ = −0.35 e Å^−3^

### 

Data collection: *PAL ADSC Quantum-210 ADX* (Arvai & Nielsen, 1983 ▶); cell refinement: *HKL3000sm* (Otwinowski & Minor, 1997 ▶); data reduction: *HKL3000sm*; program(s) used to solve structure: *SHELXL2013* (Sheldrick, 2008 ▶); program(s) used to refine structure: *SHELXL2013*; molecular graphics: *DIAMOND* (Brandenburg, 2007 ▶); software used to prepare material for publication: *WinGX* (Farrugia, 2012 ▶).

## Supplementary Material

Crystal structure: contains datablock(s) I. DOI: 10.1107/S1600536813027232/tk5261sup1.cif

Structure factors: contains datablock(s) I. DOI: 10.1107/S1600536813027232/tk5261Isup2.hkl

Supplementary material file. DOI: 10.1107/S1600536813027232/tk5261Isup3.cml

Additional supplementary materials:  crystallographic information; 3D view; checkCIF report

## Figures and Tables

**Table 1:** Hydrogen-bond geometry (Å, °)

*D*—H⋯*A*	*D*—H	H⋯*A*	*D*⋯*A*	*D*—H⋯*A*
N1—H1*N*1⋯Cl1	0.909 (13)	2.182 (14)	3.0900 (9)	177.3 (12)
N1—H2*N*1⋯N2^i^	0.907 (13)	2.200 (13)	2.9348 (11)	137.6 (10)
N2—H1*N*2⋯O1*W* ^ii^	0.887 (13)	2.262 (13)	3.1242 (12)	164.1 (11)
O1*W*—H1*O*1⋯Cl1	0.833 (19)	2.400 (19)	3.2329 (14)	178.6 (17)
O1*W*—H2*O*1⋯Cl1^iii^	0.820 (18)	2.335 (18)	3.1479 (10)	171.1 (15)
O2*W*—H1*O*2⋯O1*W* ^iv^	0.84 (2)	2.06 (2)	2.9021 (15)	178.1 (18)

Symmetry codes: (i) 
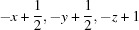
; (ii) 
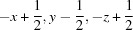
; (iii) 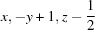; (iv) 
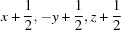
.

**Fig. 1.**
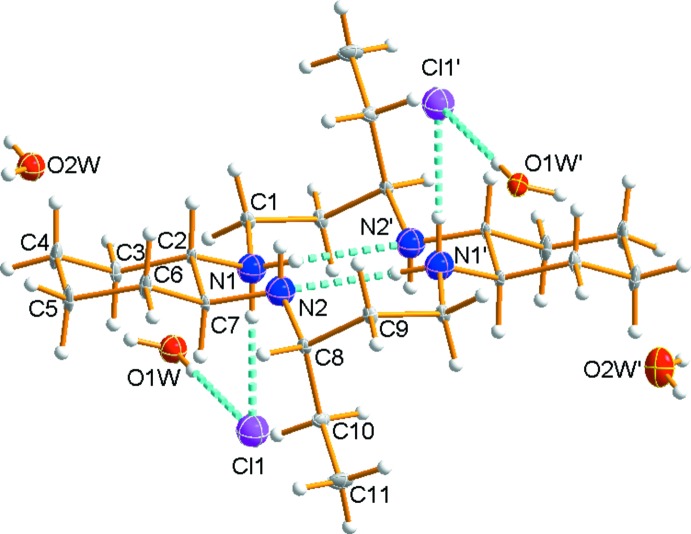
The molecular structure of title compound with displacements ellipsoids drawn at the 50% probability level for non-H atoms. Primed atoms are related by the symmetry operation 1/2-x, 1/2-y, 1-z.

## supplementary crystallographic information

### 1. Comment

The coordination chemistry of tetraazamacrocycles with steric hindrance on the macrocyclic ring, and their complexes are of interest because of their various applications (Choi *et al.*, 2010). Recently, the constrained cyclam derivatives have been reported to exhibit anti-HIV effects and to stimulate the activity of stem cells from the bone marrow (De Clercq, 2010).

The title compound, Fig. 1, containing a positively charged macrocycle, Cl^-^ and water molecules was characterized during the studies of di-*N*-substituted macrocyclic ligands as well as their corresponding copper(II) complexes. The macrocylic ligand lies on a center-of-inversion. Thus, the asymmetric unit contains half of a macrocylic dication, one chloride anion and two water molecules. The four N atoms are coplanar, and the ethyl substituents are *anti* with respect to the macrocyclic plane as a result of the symmetry of the molecule. The C—C and C—N lengths and associated angles are in the normal range (Choi *et al.*, 2006, 2011). As expected, the N–C distances involving the protonated nitrogen atom, N1 are slightly longer than the other N–C distances. The cyclohexane ring that is fused to the 14-membered ring exists in a stable chair conformation, and the N1—C2—C3—N2 torsion angle displays a *gauche* conformation. The crystal structure is stabilized by different types of hydrogen bonds, Table 1.

### 2. Experimental

The starting material, the macrocycle 3,14-diethyl-2,6,13,17-tetraazatricyclo(16.4.0.0^7,12^)docosane (L) was prepared according to published procedure (Lim *et al.*, 2006). L (0.67 g, 0.2 mmol) was taken in a round bottomed flask in EtOH (10 ml). 2-Chloro-*N*,*N*-diethylacetamide (0.0936 g, 0.5 mmol) in EtOH (5 ml) was added. Then triethylamine (1.33 g, 0.2 mmol) in EtOH (2 ml) was added. The mixture was heated to reflux for 24 h. Colourless crystals suitable for X-ray analysis were obtained from the solution at 298 K over a period of a few days.

### 3. Refinement

The C-bound H-atoms were placed in calculated positions (C—H = 0.98–1.00 Å) and were included in the refinement in the riding model approximation with *U*_iso_(H) set to 1.2–1.5*U*_eq_(C). The O- and N-bound H-atoms were located in a difference Fourier map and refined freely. One of the H atoms of the O2w water molecule was disordered over two sites of equal weight.

### Crystal data

**Table:**

C_22_H_46_N_4_^2+^·2Cl^−^·4H_2_O	*F*(000) = 1120
*M**_r_* = 509.59	*D*_x_ = 1.176 Mg m^−^^3^
Monoclinic, *C*2/*c*	Synchrotron radiation, λ = 0.72000 Å
*a* = 22.122 (4) Å	Cell parameters from 31113 reflections
*b* = 13.616 (3) Å	θ = 1.3–66.4°
*c* = 10.565 (2) Å	µ = 0.27 mm^−^^1^
β = 115.23 (3)°	*T* = 95 K
*V* = 2878.5 (10) Å^3^	Block, colourless
*Z* = 4	0.31 × 0.28 × 0.25 mm

### Data collection

**Table:**

ADSC Q210 CCD area-detector diffractometer	3663 independent reflections
Radiation source: PLSII 2D bending magnet	3446 reflections with *I* > 2σ(*I*)
Si(111) double crystal monochromator	*R*_int_ = 0.028
ω scan	θ_max_ = 29.0°, θ_min_ = 1.8°
Absorption correction: empirical (using intensity measurements) (*HKL-3000 *SCALEPACK**; Otwinowski & Minor, 1997)	*h* = −29→29
*T*_min_ = 0.922, *T*_max_ = 0.937	*k* = −18→18
13046 measured reflections	*l* = −14→14

### Refinement

**Table:**

Refinement on *F*^2^	Primary atom site location: structure-invariant direct methods
Least-squares matrix: full	Secondary atom site location: difference Fourier map
*R*[*F*^2^ > 2σ(*F*^2^)] = 0.029	Hydrogen site location: inferred from neighbouring sites
*wR*(*F*^2^) = 0.079	H atoms treated by a mixture of independent and constrained refinement
*S* = 1.05	*w* = 1/[σ^2^(*F*_o_^2^) + (0.0412*P*)^2^ + 2.157*P*] where *P* = (*F*_o_^2^ + 2*F*_c_^2^)/3
3663 reflections	(Δ/σ)_max_ < 0.001
178 parameters	Δρ_max_ = 0.46 e Å^−^^3^
0 restraints	Δρ_min_ = −0.35 e Å^−^^3^

### Special details

**Table:**

Geometry. All e.s.d.'s (except the e.s.d. in the dihedral angle between two l.s. planes) are estimated using the full covariance matrix. The cell e.s.d.'s are taken into account individually in the estimation of e.s.d.'s in distances, angles and torsion angles; correlations between e.s.d.'s in cell parameters are only used when they are defined by crystal symmetry. An approximate (isotropic) treatment of cell e.s.d.'s is used for estimating e.s.d.'s involving l.s. planes.
Refinement. Refinement of *F*^2^ against ALL reflections. The weighted *R*-factor *wR* and goodness of fit *S* are based on *F*^2^, conventional *R*-factors *R* are based on *F*, with *F* set to zero for negative *F*^2^. The threshold expression of *F*^2^ > σ(*F*^2^) is used only for calculating *R*-factors(gt) *etc*. and is not relevant to the choice of reflections for refinement. *R*-factors based on *F*^2^ are statistically about twice as large as those based on *F*, and *R*- factors based on ALL data will be even larger.

### Fractional atomic coordinates and isotropic or equivalent isotropic displacement parameters (Å^2^)

**Table:**

	*x*	*y*	*z*	*U*_iso_*/*U*_eq_	Occ. (<1)
N1	0.24338 (3)	0.30241 (5)	0.30460 (7)	0.00545 (13)	
H1N1	0.2484 (6)	0.3678 (10)	0.3234 (13)	0.013 (3)*	
H2N1	0.2370 (6)	0.2756 (9)	0.3766 (13)	0.014 (3)*	
N2	0.34493 (3)	0.23871 (5)	0.55886 (7)	0.00584 (14)	
H1N2	0.3460 (6)	0.1738 (10)	0.5533 (13)	0.012 (3)*	
C1	0.18207 (4)	0.28405 (6)	0.17163 (8)	0.00738 (15)	
H1A	0.1831	0.2160	0.1398	0.009*	
H1B	0.1819	0.3293	0.0981	0.009*	
C2	0.30641 (4)	0.26138 (6)	0.30527 (8)	0.00612 (15)	
H2	0.3004	0.1891	0.2872	0.007*	
C3	0.32229 (4)	0.30822 (7)	0.19167 (8)	0.01049 (16)	
H3A	0.3289	0.3798	0.2083	0.013*	
H3B	0.2844	0.2980	0.0990	0.013*	
C4	0.38574 (4)	0.26195 (8)	0.19333 (9)	0.01451 (18)	
H4A	0.3775	0.1916	0.1680	0.017*	
H4B	0.3971	0.2947	0.1226	0.017*	
C5	0.44453 (4)	0.27145 (8)	0.33736 (9)	0.01465 (18)	
H5A	0.4836	0.2358	0.3377	0.018*	
H5B	0.4569	0.3415	0.3572	0.018*	
C6	0.42661 (4)	0.22968 (7)	0.45105 (9)	0.01216 (17)	
H6A	0.4645	0.2403	0.5436	0.015*	
H6B	0.4194	0.1580	0.4370	0.015*	
C7	0.36367 (4)	0.27744 (6)	0.44987 (8)	0.00632 (15)	
H7	0.3718	0.3497	0.4651	0.008*	
C8	0.39051 (4)	0.27036 (6)	0.70259 (8)	0.00656 (15)	
H8	0.4375	0.2635	0.7134	0.008*	
C9	0.38146 (4)	0.20090 (6)	0.80803 (8)	0.00776 (15)	
H9A	0.3820	0.1324	0.7770	0.009*	
H9B	0.4203	0.2089	0.8998	0.009*	
C10	0.37854 (4)	0.37876 (6)	0.72406 (9)	0.01032 (16)	
H10A	0.3825	0.4182	0.6492	0.012*	
H10B	0.3324	0.3865	0.7153	0.012*	
C11	0.42749 (5)	0.41859 (7)	0.86611 (10)	0.01776 (19)	
H11A	0.4227	0.3815	0.9408	0.027*	
H11B	0.4179	0.4881	0.8734	0.027*	
H11C	0.4733	0.4117	0.8753	0.027*	
Cl1	0.256487 (11)	0.526092 (15)	0.35905 (2)	0.01378 (8)	
O1W	0.16965 (4)	0.51673 (5)	0.02378 (8)	0.01737 (15)	
H1O1	0.1926 (9)	0.5195 (12)	0.110 (2)	0.038 (4)*	
H2O1	0.1952 (8)	0.5022 (12)	−0.0111 (17)	0.033 (4)*	
O2W	0.52804 (5)	0.02912 (8)	0.40229 (12)	0.0354 (2)	
H1O2	0.5693 (11)	0.0172 (13)	0.4382 (19)	0.044 (5)*	
H2O2	0.512 (2)	0.016 (3)	0.328 (5)	0.062 (14)*	0.50
H3O2	0.5140 (19)	0.015 (3)	0.457 (4)	0.041 (10)*	0.50

### Atomic displacement parameters (Å^2^)

**Table:**

	*U*^11^	*U*^22^	*U*^33^	*U*^12^	*U*^13^	*U*^23^
N1	0.0030 (3)	0.0080 (3)	0.0048 (3)	−0.0006 (2)	0.0011 (2)	−0.0001 (2)
N2	0.0045 (3)	0.0083 (3)	0.0045 (3)	−0.0011 (2)	0.0017 (2)	−0.0001 (2)
C1	0.0037 (3)	0.0124 (4)	0.0044 (3)	−0.0012 (3)	0.0001 (3)	0.0005 (3)
C2	0.0030 (3)	0.0095 (3)	0.0061 (3)	0.0000 (3)	0.0021 (3)	−0.0009 (3)
C3	0.0072 (4)	0.0190 (4)	0.0057 (3)	−0.0013 (3)	0.0032 (3)	0.0007 (3)
C4	0.0084 (4)	0.0291 (5)	0.0080 (4)	−0.0005 (3)	0.0054 (3)	−0.0023 (3)
C5	0.0058 (4)	0.0308 (5)	0.0093 (4)	−0.0008 (3)	0.0050 (3)	−0.0001 (3)
C6	0.0050 (3)	0.0232 (4)	0.0093 (4)	0.0032 (3)	0.0040 (3)	0.0019 (3)
C7	0.0032 (3)	0.0109 (4)	0.0049 (3)	−0.0011 (3)	0.0018 (3)	0.0000 (3)
C8	0.0036 (3)	0.0099 (4)	0.0053 (3)	−0.0008 (3)	0.0010 (3)	−0.0002 (3)
C9	0.0041 (3)	0.0111 (4)	0.0069 (3)	0.0010 (3)	0.0012 (3)	0.0023 (3)
C10	0.0131 (4)	0.0091 (4)	0.0081 (3)	−0.0011 (3)	0.0039 (3)	−0.0007 (3)
C11	0.0209 (5)	0.0151 (4)	0.0129 (4)	−0.0049 (4)	0.0030 (3)	−0.0055 (3)
Cl1	0.01970 (12)	0.00834 (11)	0.01508 (12)	−0.00076 (7)	0.00912 (9)	0.00064 (7)
O1W	0.0187 (3)	0.0182 (3)	0.0168 (3)	0.0023 (3)	0.0092 (3)	0.0010 (3)
O2W	0.0226 (5)	0.0488 (6)	0.0330 (5)	0.0101 (4)	0.0101 (4)	0.0081 (4)

### Geometric parameters (Å, º)

**Table:**

N1—C2	1.4995 (10)	C6—C7	1.5320 (11)
N1—C1	1.5003 (12)	C6—H6A	0.9900
N1—H1N1	0.909 (13)	C6—H6B	0.9900
N1—H2N1	0.907 (13)	C7—H7	1.0000
N2—C7	1.4774 (10)	C8—C10	1.5333 (12)
N2—C8	1.4842 (11)	C8—C9	1.5381 (11)
N2—H1N2	0.887 (13)	C8—H8	1.0000
C1—C9^i^	1.5234 (11)	C9—C1^i^	1.5233 (11)
C1—H1A	0.9900	C9—H9A	0.9900
C1—H1B	0.9900	C9—H9B	0.9900
C2—C3	1.5264 (11)	C10—C11	1.5276 (13)
C2—C7	1.5285 (12)	C10—H10A	0.9900
C2—H2	1.0000	C10—H10B	0.9900
C3—C4	1.5318 (12)	C11—H11A	0.9800
C3—H3A	0.9900	C11—H11B	0.9800
C3—H3B	0.9900	C11—H11C	0.9800
C4—C5	1.5286 (13)	O1W—H1O1	0.833 (19)
C4—H4A	0.9900	O1W—H2O1	0.820 (18)
C4—H4B	0.9900	O2W—H1O2	0.84 (2)
C5—C6	1.5262 (12)	O2W—H2O2	0.73 (4)
C5—H5A	0.9900	O2W—H3O2	0.78 (4)
C5—H5B	0.9900		
			
C2—N1—C1	114.20 (6)	C5—C6—H6A	109.2
C2—N1—H1N1	109.7 (8)	C7—C6—H6A	109.2
C1—N1—H1N1	110.2 (8)	C5—C6—H6B	109.2
C2—N1—H2N1	108.8 (8)	C7—C6—H6B	109.2
C1—N1—H2N1	108.5 (8)	H6A—C6—H6B	107.9
H1N1—N1—H2N1	105.0 (11)	N2—C7—C2	109.85 (6)
C7—N2—C8	113.56 (6)	N2—C7—C6	113.46 (7)
C7—N2—H1N2	106.2 (8)	C2—C7—C6	108.08 (7)
C8—N2—H1N2	109.4 (8)	N2—C7—H7	108.4
N1—C1—C9^i^	111.44 (7)	C2—C7—H7	108.4
N1—C1—H1A	109.3	C6—C7—H7	108.4
C9^i^—C1—H1A	109.3	N2—C8—C10	110.24 (6)
N1—C1—H1B	109.3	N2—C8—C9	108.72 (6)
C9^i^—C1—H1B	109.3	C10—C8—C9	113.61 (7)
H1A—C1—H1B	108.0	N2—C8—H8	108.0
N1—C2—C3	111.46 (7)	C10—C8—H8	108.0
N1—C2—C7	108.91 (7)	C9—C8—H8	108.0
C3—C2—C7	110.83 (7)	C1^i^—C9—C8	115.61 (7)
N1—C2—H2	108.5	C1^i^—C9—H9A	108.4
C3—C2—H2	108.5	C8—C9—H9A	108.4
C7—C2—H2	108.5	C1^i^—C9—H9B	108.4
C2—C3—C4	109.63 (7)	C8—C9—H9B	108.4
C2—C3—H3A	109.7	H9A—C9—H9B	107.4
C4—C3—H3A	109.7	C11—C10—C8	113.10 (7)
C2—C3—H3B	109.7	C11—C10—H10A	109.0
C4—C3—H3B	109.7	C8—C10—H10A	109.0
H3A—C3—H3B	108.2	C11—C10—H10B	109.0
C5—C4—C3	111.30 (7)	C8—C10—H10B	109.0
C5—C4—H4A	109.4	H10A—C10—H10B	107.8
C3—C4—H4A	109.4	C10—C11—H11A	109.5
C5—C4—H4B	109.4	C10—C11—H11B	109.5
C3—C4—H4B	109.4	H11A—C11—H11B	109.5
H4A—C4—H4B	108.0	C10—C11—H11C	109.5
C6—C5—C4	110.83 (7)	H11A—C11—H11C	109.5
C6—C5—H5A	109.5	H11B—C11—H11C	109.5
C4—C5—H5A	109.5	H1O1—O1W—H2O1	106.6 (16)
C6—C5—H5B	109.5	H1O2—O2W—H2O2	111 (4)
C4—C5—H5B	109.5	H1O2—O2W—H3O2	108 (3)
H5A—C5—H5B	108.1	H2O2—O2W—H3O2	124 (5)
C5—C6—C7	112.06 (7)		
			
C2—N1—C1—C9^i^	162.93 (7)	C3—C2—C7—N2	175.38 (6)
C1—N1—C2—C3	62.08 (9)	N1—C2—C7—C6	176.69 (7)
C1—N1—C2—C7	−175.33 (6)	C3—C2—C7—C6	−60.34 (9)
N1—C2—C3—C4	−178.28 (7)	C5—C6—C7—N2	−179.94 (7)
C7—C2—C3—C4	60.24 (9)	C5—C6—C7—C2	57.97 (9)
C2—C3—C4—C5	−56.36 (10)	C7—N2—C8—C10	73.59 (8)
C3—C4—C5—C6	54.06 (11)	C7—N2—C8—C9	−161.26 (6)
C4—C5—C6—C7	−55.52 (11)	N2—C8—C9—C1^i^	−75.02 (9)
C8—N2—C7—C2	−167.13 (6)	C10—C8—C9—C1^i^	48.13 (9)
C8—N2—C7—C6	71.78 (9)	N2—C8—C10—C11	−175.53 (7)
N1—C2—C7—N2	52.42 (8)	C9—C8—C10—C11	62.15 (9)

Symmetry code: (i) −*x*+1/2, −*y*+1/2, −*z*+1.

### Hydrogen-bond geometry (Å, º)

**Table:**

*D*—H···*A*	*D*—H	H···*A*	*D*···*A*	*D*—H···*A*
N1—H1*N*1···Cl1	0.909 (13)	2.182 (14)	3.0900 (9)	177.3 (12)
N1—H2*N*1···N2^i^	0.907 (13)	2.200 (13)	2.9348 (11)	137.6 (10)
N2—H1*N*2···O1*W*^ii^	0.887 (13)	2.262 (13)	3.1242 (12)	164.1 (11)
O1*W*—H1*O*1···Cl1	0.833 (19)	2.400 (19)	3.2329 (14)	178.6 (17)
O1*W*—H2*O*1···Cl1^iii^	0.820 (18)	2.335 (18)	3.1479 (10)	171.1 (15)
O2*W*—H1*O*2···O1*W*^iv^	0.84 (2)	2.06 (2)	2.9021 (15)	178.1 (18)

Symmetry codes: (i) −*x*+1/2, −*y*+1/2, −*z*+1; (ii) −*x*+1/2, *y*−1/2, −*z*+1/2; (iii) *x*, −*y*+1, *z*−1/2; (iv) *x*+1/2, −*y*+1/2, *z*+1/2.
